# Antibiotic use practices of veterinarians and para-veterinarians and the implications for antibiotic stewardship in Nigeria

**DOI:** 10.4102/jsava.v92i0.2120

**Published:** 2021-05-28

**Authors:** Adah Ogwuche, Abel B. Ekiri, Isabella Endacott, Beatty-Viv Maikai, Enokela S. Idoga, Ruth Alafiatayo, Alasdair J.C. Cook

**Affiliations:** 1 Zoetis-ALPHA Initiative, Zoetis, Zaventem, Belgium; 2 Department of Veterinary Epidemiology and Public Health, School of Veterinary Medicine, Faculty of Health and Medical Sciences, University of Surrey, Guildford, United Kingdom; 3 Department of Veterinary Public Health and Preventative Medicine, Faculty of Veterinary Medicine, Ahmadu Bello University, Zaria, Nigeria; 4 Department of Veterinary Physiology, Biochemistry and Pharmacology, Faculty of Health Sciences, University of Jos, Jos, Nigeria

**Keywords:** antibiotic, veterinarian, para-veterinarian, animal health, Nigeria, antibiotic stewardship, antimicrobial resistance

## Abstract

The aim of this study was to describe the antibiotic use practices of veterinarians and para-veterinarians in Nigeria. An online survey was distributed during November through December 2018 via email and phone to veterinarians and para-veterinarians to collect information on antibiotic use practices. Data were downloaded into Excel and descriptive statistics were presented and analysed. The survey was completed by 390 respondents. Almost all respondents (98.5%, 384/390) recommended the use of antibiotics to treat animal patients, and of these, 93.2% (358/384) were veterinarians and 6.8% (26/384) were para-veterinarians. Most respondents reported commonly recommending the use of oxytetracycline (82.6%, 317/384), tylosin (44.5%, 171/384) and gentamycin (43.8%, 168/384). A third (32.0%, 122/384) of respondents did not undertake antimicrobial susceptibility testing (AST) prior to antibiotic treatment. At least 60% of the respondents recommended the use of antibiotics for the treatment of non-bacterial pathogens, including viral, helminth and fungal pathogens. Over 55% (217/390) were not aware of government-issued guidelines on antibiotic use in animals, although of those aware, 69% (74/107) utilised the guidelines. Across all respondents, the majority believed legislation or regulation by government can influence the use of antibiotics by animal health professionals. The study highlights areas that can be targeted as part of intervention strategies to promote antimicrobial stewardship by animal health professionals in Nigeria, including the need for increased use of AST as a tool for supporting disease management, increased awareness of appropriate antibiotic use and greater dissemination of antibiotic use guidelines and enforcement of relevant regulation by government authorities.

## Introduction

The therapeutic benefits of antibiotics to humans, livestock and companion animals are well recognised (Ventola 2015). In livestock, antibiotics are essential in the therapeutic treatment of bacterial diseases that impact livestock health, production and welfare (Van et al. 2020). Antibiotics are also used for prophylaxis and metaphylaxis, to maintain animal health and increase productivity. In companion animals, antimicrobials are essential for therapeutic treatment of skin, wound, respiratory and urinary tract infections, as well as for reducing the frequency of sepsis and surgical site infections (Vasseur et al. 1988). However, despite the huge successes recorded against bacteria, recent trends have shown a decline in the ability of antibiotics to control pathogenic bacteria (Richardson 2017). Bacteria are becoming increasingly resistant to antibiotics, posing potential risks to veterinary health, welfare, food and feed production systems (FAO 2016), and in humans, resulting in treatment failures and leading to increased morbidity, higher medical costs, prolonged hospital stays for human cases and increased mortality (WHO 2008).

Antibiotic resistance is accelerated by the overuse and misuse of antibiotics (WHO 2008). The use of antibiotics exerts selection pressure on microbes, allowing resistant pathogens to proliferate and leading to the emergence of antimicrobial resistance (AMR). The emergence of AMR in humans has been linked with AMR in animals and the environment (Forsberg et al. 2012; Mather et al. 2013; Spoor et al. 2013; Ward et al. 2014). Interaction between humans, animals and the environment helps to promote the transfer of resistance genes across different species, with potential bidirectional transfer of resistance genes between human and animal species (Fernandes et al. 2018), making AMR an important One Health challenge emerging on a global scale (Forsberg et al. 2012; Mather et al. 2013; Spoor et al. 2013; Van den Bogaard et al. 2001; Ward et al. 2014). With the dwindling repertoire of antibiotic options available for the control of emerging, hard-to-treat and multidrug-resistant bacteria, there is need for proper antibiotic stewardship to preserve the efficacy of existing antibiotics (Aslam et al. 2018).

The global consumption of antibiotics used in food animals during 2010–2030 is predicted to rise by 67%, with the bulk of this increased antibiotic use attributed to low- and middle-income countries (Van Boeckel et al. 2015). Nigeria was listed amongst the top 50 consumers of antibiotics in food animals in 2010 and is projected to increase food animal antibiotic use by 167% by 2030 (Manyi-Loh et al. 2018). The expanding population and rising income levels in low- and middle-income countries have increased the demand for animal protein and, combined with a gradual shift towards intensive farming systems, might lead to increased use of antimicrobials (Manyi-Loh et al. 2018). As an example, Nigeria is anticipated to become the third most populated country in the world by 2050 with production in the poultry, fishing and cattle industries intensifying to meet the demands for food security and alleviate poverty and malnutrition (WHO 2018).

In Nigeria, veterinarians and para-veterinarians are responsible for prescribing and administering antibiotics in both companion and food animals, as in most sub-Saharan African countries. Factors that influence decisions on antibiotic use can vary and include training, published literature, written guidelines and personal experience or anecdotal practices (De Briyne et al. 2013), as well as type of veterinary practice (Norris et al. 2019). Understanding the antibiotic use practices of veterinarians and para-veterinarians can bridge a significant knowledge gap by helping to identify the most frequently used antibiotics, patterns and reasons behind their usage, and the extent to which veterinarians employ diagnostics in making therapeutic decisions that influence antibiotic use. Such information would help identify areas that can be targeted when developing guidelines for prudent antibiotic use in veterinary practice in Nigeria and subsequently assist policy formulators in making informed decisions to promote antimicrobial stewardship (AMS). The objective of this study was to characterise the antibiotic use practices of veterinarians and para-veterinarians in Nigeria.

## Methods

A self-administered survey was designed to collect information on the demographics and antibiotic-prescribing habits of veterinarians and para-veterinarians. The survey was pretested amongst five veterinarians in Nigeria and further refined before finalisation. The finalised survey was administered between 27 November 2018 and December 2018 by using the Qualtrics platform and respondents contacted via the social media platforms (WhatsApp, Facebook, Telegram) of various state Veterinary and Para-Veterinary associations.

In the current study setting, use of the phrase ‘prescribing antibiotics’ does not fully reflect the different ways animal owners may get access to antibiotics used to treat animals and the role of veterinary professionals and animal owners in this process. In this study setting, as well as in other sub-Saharan Africa country settings, typically, veterinary professionals recommend the use of antibiotics to treat animal patients without issuing written prescriptions that can be presented to a pharmacy prior to purchase of antibiotics. The recommended antibiotics can be purchased by the animal owner from the veterinary professional; either directly from the veterinarian’s ambulatory drug stock or from the veterinarian’s drug shop or pharmacy. Alternatively, the animal owner can purchase from another available drug shop or pharmacy. Antibiotic drug administration is usually performed by either the veterinarian or the animal owner, depending on the arrangements between the veterinarian and animal owner. It is also important to note that in the current study setting, animal owners can purchase and use antibiotics to treat their animals with or without a recommendation from a qualified veterinary professional. Therefore, for purposes of this study, use of the phrase ‘recommending use of antibiotics’ for animal patients was considered more encompassing and appropriate and used throughout the article (vs. prescribing antibiotics).

### Study area and population

The target respondents were veterinarians and para-veterinarians practising in Nigeria. A veterinarian was defined as any individual with a Doctor of Veterinary Medicine (DVM) degree from any of the veterinary faculties in the country or an expatriate veterinarian, certified by the Veterinary Council of Nigeria (VCN) to practise veterinary medicine in Nigeria. The VCN is the body authorised by law to issue practice licenses and to regulate all practising veterinarians in Nigeria. The total number of registered veterinarians in Nigeria stands at 9213, including those who have died, living in diaspora, or not practising (Personal Communication, College secretary, College of Veterinary Surgeons, Abuja, Nigeria). However, the total number of veterinarians with updated practice licenses (assumed to be those in active practice in 2019) stands at about 3500. It is estimated that more than 2500 of the active veterinarians are young veterinarians, who graduated in the last 15–20 years (Personal Communication, College secretary, College of Veterinary Surgeons, Abuja, Nigeria), accounting for over 70% of the prescribing population. Most of the older veterinarians are in government employment and less likely to be active field practitioners prescribing antibiotics (personal communication, College secretary, College of Veterinary Surgeons, Nigeria). A Para-Veterinarian was defined as a veterinary assistant/technologist with an ordinary national diploma (OND) in animal health or higher national diploma (HND) from any of the country’s colleges of agriculture or college of animal health technology. ‘Para-veterinarian’ is sometimes also expanded to include extension workers/individuals with qualifications below an OND or HND who have received specific trainings or are skilled in any area of animal health or animal health-supporting services. Specific para-veterinary skills may include (but not limited to) disease diagnosis and treatment, laboratory technology, animal restraint, hoof trimming, artificial insemination, dairy management and general animal production. In most instances, however, para-veterinarians are not provided in-depth training in pharmacology, and although some may have received some basic training on responsible antibiotic use, most are not likely to be knowledgeable about antibiotic stewardship. Many para-veterinarians rely on what they have watched veterinarians do over time and are likely to repeat same without knowledge as to why certain procedures are performed or why specific drugs may be given in certain instance. Without an umbrella body or standard qualification for para-veterinarians, there are no official figures or estimate for the population of para-veterinarians in the animal health value chain in Nigeria. In the context of Nigeria, although considered illegal, para-veterinarians can prescribe antibiotics and recommend to farmers what antibiotics to purchase over the counter.

The Nigerian Veterinary Medical Association (NVMA) is a national body that brings together all registered veterinarians in Nigeria. It is the umbrella of all veterinary surgeons who are registered with the VCN. All licensed and practising veterinarians in Nigeria are required by law to be paying members of the NVMA. Nigeria is administratively divided into 36 states and a Federal Capital Territory. Each of these 37 governing units has local chapters of the NVMA. All veterinarians who were members of the national association and the state-level chapters were targeted via chat groups. Such chat groups are normally hosted on social media platforms (and these included WhatsApp, Facebook and Telegram) and used as a formal communication and interaction channel between veterinary and para-veterinary practitioners. These platforms are used to pass across important information and deliberate on pressing issues related to veterinary practice. Veterinarians were also encouraged to share the survey link with para-veterinary colleagues in each state. Participation was limited to veterinarians or para-veterinarians with the access and ability to carry out Internet-related functions on an android device, iPhone operation system (iOS) device or tablet or a laptop. It was also a requirement for a participant to be active on social media platforms such as WhatsApp, Facebook and Telegram or own an active and accessible email account. Veterinarians and para-veterinarians who did not meet these inclusion criteria were by default unable to participate in the survey.

### Sample size

A non-probabilistic sampling method was adopted. Study participants were identified based on convenience as those within the identified group who had access to android phones, iOS devices, tablets or laptops and had active social media or email accounts. In Nigeria, approximately, 13% of the general population is thought to own a smartphone (Newzoo 2018), but this number is likely to be higher amongst the veterinarian and para-veterinarian professional group because generally Nigerians with tertiary education have a higher income and exposure to information technology compared with the general population (Adetimirin 2011; The World Bank 2015). The proportions of veterinarians and para-veterinarians who own smartphones were unknown, and eligible veterinarians and para-veterinarians may have been excluded because they did not own or have access to a smartphone or tablet or laptop.

### Approach to survey administration

The questionnaire was administered online in English language to veterinary and para-veterinary professionals by using social media platforms. In Africa, the rapidly growing rate of social media patronage is one of the fastest globally (Dahir 2018). Most organisations, companies and associations rely on social media chat groups to disseminate information. WhatsApp and Facebook are the most frequently used social media applications with over 191 million active users across Africa (Dahir 2018). Many veterinarians belong to WhatsApp groups maintained by their respective state veterinary associations, which are mostly used for information dissemination, knowledge sharing and discussion. Several other para-veterinary associations also have WhatsApp groups scattered across the country.

### Data collection

The questionnaire tool collected data on demographics, antibiotic use practices, laboratory use and antibiotic usage regulations (Online Appendix 1 - Questionnaire). The specific target audience (veterinary and para-veterinary professionals) were reached by sending a survey link to focus social media groups including national associations, local state associations, faculty staff associations, ministry staff and several similar professional chat rooms and groups and to individuals on social media platforms (WhatsApp, Facebook, Telegram) and via emails. The groups were identified by contacting individual state NVMA officials and reaching out to key opinion leaders (KOLs) in the listed institutions (e.g. universities, federal ministry of agriculture and rural development and private veterinary fora) who in turn reached out to colleagues across Nigeria’s Northern and Southern divide. Respondents completed the survey via the use of phones, tablets (android or iOS) and laptops. Participation in the survey was voluntary and on opening of the survey link consent to participate was confirmed by clicking ‘I consent’ prior to survey completion. The survey was administered over a span of 4 weeks. Respondents could only participate once from the same device to prevent multiple entries from a single individual, and participants were encouraged to complete the survey once. Participants were reminded twice at the end of the first and third weeks during the 4-week survey administration period.

### Data analysis

The survey was administered by using the online survey platform Qualtrics and at completion, survey data were downloaded into Microsoft Excel (365 MSO) and descriptive statistical analysis were performed.

### Ethical considerations

Ethical review and approval were granted by the Ahmadu Bello University, Nigeria, via an ethics review application (Ethics approval number: ABUCUHSR/2018/008) and by the Research Integrity and Governance Office at the University of Surrey, United Kingdom (Response ID: 353003-352994-41441149).

## Results

The total number of participants who responded to the survey was 416; 413/416 respondents completed the survey; and 3/416 attempted but did not complete the survey. The response rate for veterinarians was estimated at 10.3% (362/3500), calculated as the number of veterinarians who responded divided by the estimated total number of veterinarians with updated practice licenses in Nigeria. The response rate for para-veterinarians was not estimated because there are no official figures or estimate for the population of para-veterinarians involved in the animal health value chain in Nigeria.

### Demographic information

A total of 413 participants completed the survey. Most of the respondents (87.7%, 362/413) were veterinarians; 6.8% (28/413) were para-veterinarians; 2.9% (12/413) identified themselves as farmers or farm managers; and another 2.4% (10/413) identified as ‘other’ and did not fit into any of the listed categories. These 10 respondents identified as 4 animal scientists, 3 microbiologists, 2 veterinary students and 1 veterinary pharmaceutical manager (Table 1). One additional respondent identified as ‘farmer and veterinarian and para-veterinarian and farm manager’.

**TABLE 1:** Demographics of study participants.

Variable	Response	Respondents	
		*n*	%
Profession **(*n* = 413)**	Veterinarian	362	87.7
	Para-veterinarian	28	6.8
	Farmer or farm manager‡	12	2.9
	Other‡	10	2.4
	Veterinarian + Para-veterinarian + Farmer + Farm manager‡	1	0.2
Highest level of education (*n* = 390)	Veterinary degree (DVM/BVS)	216	55.4
	DVM/BVS + MSc/MPH/PhD minimum	137	35.1
	BSc/HND + MSc/MPH/PhD minimum	14	3.6
	Bachelor’s degree (BSc)	9	2.3
	Other	9	2.3
	Diploma (OND)/NCE	5	1.3
Age group (years) (*n* = 390)	26–35	225	57.7
	36–45	62	15.9
	Over 45	61	15.6
	25 or less	38	9.7
	Prefer not to say	4	1.0
Employment status† (*n* = 390)	Private practice	207	53.1
	Government employee	136	34.9
	Research	53	13.6
	Teaching	42	10.8
	Non-governmental organisation (NGO) employee	41	10.5
	Other	11	2.8
Profession experience (years) (*n* = 390)	0–5	188	48.2
	6–10	107	27.4
	11 or more	91	23.3
	Prefer not to say	4	1.0

DVM, Doctor of Veterinary Medicine; BVS, Bachelor of Veterinary Sciences; BSc, Bachelor of Science; MSc, Master of Science; MPH, Master of Public Health; PhD, Doctor of Philosophy; OND, Ordinary National Diploma; NCE, Nigeria Certificate in Education; HND, higher national diploma.

† , All applicable responses were selected. Survey respondents were invited to select all applicable responses and as such the respondent total may not add up to 100% for these variables.

‡ , Respondents did not meet study criteria and were hence excluded from all other analyses. A total of 23/413 respondents were excluded.

A total of 23/413 participants were excluded from the analysis because the main respondents of interest in this study were veterinarians and para-veterinarians only. In Nigeria, veterinarians and para-veterinarians can work as farmers (may own their own farms) or as farm managers (either manage their own farms or hired to manage somebody else’s farm), and so the respondents were given the option to also select these two options (farmer or farm manager) in this survey question. However, it was difficult to determine and confirm if the respondents who identified as ‘farmer’ and ‘farmer manager’ (*n* = 12/413) or as ‘others’ (*n* = 10/413) were additionally veterinarians or para-veterinarians and so were dropped from further analysis. The one respondent who was identified as ‘farmer and veterinarian and para-veterinarian and farm manager’ (*n* = 1/413) was also dropped from the analysis because it was difficult to clearly determine if the respondent was a veterinarian or para-veterinarian. The remaining 390 respondents (362 veterinarians and 28 para-veterinarians) were considered for further analyses.

Most of the respondents were 26–35 years old (225/390 [57.7%]).With respect to category of employment, over half of the respondents (207/390 [53.1%]) were involved in private practice, whereas 34.9% (136/390) were government employees. Most respondents (188/390 [48.2%]) had 0–5 years of veterinary work/practice experience (Table 1).

### Antibiotic use practices

Of the 390 respondents, 384 (98.5%) recommended the use of antibiotics to treat animal patients, whereas 6/390 (1.5%) did not and were excluded from further analyses. Of the 384 respondents who recommended the use of antibiotics, 93.2% (358/384) were veterinarians and 6.8% (26/384) were para-veterinarians. The employment categories for the 384 respondents who reported using antibiotics were as follows: private practice (205/384 [53.4%]), government (134/384 [34.9%]), research (52/384 [13.5%]) teaching (40/384 [10.4%]), workers within non-governmental organisations (41/384 [10.7%]) and other (11/384 [2.9%]). Chickens (260/384 [67.7%]), followed by dogs 65.4% (251/384) were the most commonly seen animal species in respondents’ clinical practices (Appendix 1 – Table 1-A1). Regarding the animal species to which practitioners most often used antibiotics, the majority of respondents (276/384 [71.9%]) used antibiotics for chicken followed by 69.3% (266/384) who reported using antibiotics for dogs (Table 2). As indicated here chickens and dogs are the most frequently seen animal species in veterinary practice as well as the animal species for which antibiotics were mostly used.

**TABLE 2:** Animal species in which the use of antibiotics was most often recommended by animal health professionals (*n* = 384).

Animal species	Respondents†	
	*n*	%
Chickens	276	71.9
Dogs	266	69.3
Goats	230	59.9
Cattle	203	52.9
Sheep	200	52.1
Turkeys	111	28.9
Cats	94	24.5
Fish/aquaculture	51	13.3
Others	11	2.9

† , All applicable responses selected.

### Antibiotics used in animal practice

When requested to report the five most frequently recommended antibiotics to treat animal patients, respondents commonly recommended the use of oxytetracycline (317/384 [82.6%]), tylosin (171/384 [44.5%]), gentamycin (168/384 [43.8%]), penicillin (151/384 [39.3%]) and enrofloxacin (148/384 [38.5%]) (Appendix 1 – Table 2-A1).

### Reasons for recommending the use of antibiotics to treat animal patients

The respondents were asked the two most common reasons for recommending the use of antibiotics to treat animal patients. Therapeutic treatment of disease (371/384 [96.6%]) was the most frequently reported reason amongst the 384 respondents who recommended the use of antibiotics, followed by disease prevention or prophylaxis (160/384 [41.7%]) and growth promotion (18/384 [4.7%]) (Table 3).

**TABLE 3:** Most common reasons for prescribing or using antibiotics in animal practice (*n* = 384).

Reason	Respondents†	
	*n*	%
Therapeutic or treatment	371	96.6
Disease prevention or prophylaxis	160	41.7
Growth promotion	18	4.7

† , All applicable responses selected.

### Type of pathogen targeted when recommending the use antibiotics to treat animals

With regard to the type of pathogens, most respondents (379/384 [98.7%]) reported targeting bacterial pathogens when recommending the use of antibiotics, and 81.3% (312/384) of respondents targeted protozoan parasites. However, the proportions of respondents who recommended the use of antibiotics in the treatment of conditions that would never respond to them including viral, helminth, fungal and other were 82.3% (316/384), 71% (273/384), 69% (265/384) and 59.3% (228/384), respectively (Appendix 1 – Table 3-A1). For veterinarians only (*n* = 358), the proportions of respondents who recommended the use of antibiotics in the treatment of bacterial, viral, protozoa, helminth, fungal and other conditions were 99.2% (355/358), 82.1% (294/358), 80.4% (288/358), 71.2% (255/358), 68.1% (244/358) and 59.5% (213/358), respectively. For para-veterinarians only (*n* = 26), the proportions of respondents who recommended the use of antibiotics in the treatment of bacterial, viral, fungal, protozoa, helminth and other conditions were 92.3% (24/26), 84.6% (22/26), 80.8% (21/26), 92.3% (24/26), 69.2% (18/26) and 57.7% (15/26), respectively.

### Source of antibiotics and source of information guiding their use

The respondents were asked the two most common sources where antibiotics were purchased or acquired. The most commonly reported sources were veterinary clinics (259/384 [67.4%]), agro-stores (177/384 [46.1%]) and distributors (154/384 [40.1%]) and open markets (60/384 [15.6%]) and 60/384 (15.6%), respectively. Other reported sources (7/384 [1.8%]) included human pharmacies and international markets (Appendix 1 – Table 4-A1, where respondents selected two most relevant responses).

The major source of information used to guide decisions on the choice of antibiotics was the manufacturer’s label on drug bottles, sachets or leaflets (289/384 respondents [75.3%]). In addition, 278/384 respondents (72.4%) used personal experience, whilst 195/384 (50.8%), 193/384 (50.3%) and 13/384 (3.4%) respondents used antibiotic guidelines developed and kept by veterinary practice, information from colleagues or others, respectively (Appendix 1 – Table 5-A1, where respondents selected three most relevant responses).

### Use of antimicrobial sensitivity testing and general use of laboratories to aid diagnosis

Almost a third of survey respondents (122/384 [31.8%]) did not undertake antimicrobial sensitivity testing before starting antibiotic treatment. Sensitivity testing was ‘frequently’ undertaken by 65/384 (16.9%) of respondents and ‘sometimes’ undertaken by 164 (42.7%) of respondents (Table 4).

**TABLE 4:** Frequency of antimicrobial sensitivity testing conducted before starting antibiotic treatment (*n* = 384).

Frequency	Respondents†	
	*n*	%
Sometimes (1–5 times/year)	164	42.7
Never	122	31.8
Frequently (> 5 times/year)	65	16.9
Don’t know	33	8.6

A third of the respondents (126/384 [32.8%]) reported they had not used laboratories to aid diagnosis in the previous year; 84/384 (21.9%) used laboratories 1–2 times; 48/384 (12.5%) used laboratories 3–4 times; and 19/384 (4.9%) used laboratories 5–6 times. Under a third of the respondents (107/384 [27.9%]) used laboratories more than 6 times in the previous year to aid diagnosis.

A total of 258 respondents reported to have used a diagnostic laboratory at some point in the previous year. Many respondents who used the laboratory submitted samples for microbiological culture and antimicrobial sensitivity testing (236/258 [91.5%]), whilst 102/258 (39.5%) requested microbiological culture and identification (without sensitivity testing). Pathology or post-mortem examination was requested by 168/258 respondents (65.1%), whilst 73/258 (28.3%) requested serology testing. Other (7/258 [2.7%]) selected tests included complete blood count (1/258 [0.4%]), haematology (4/258 [1.6%]), haematology and biochemistry (1/258 [0.4%]), polymerase chain reaction (PCR) (1/258 [0.4%]) and parasitology (1/258 [0.4%]), respectively (Appendix 1 – Table 6-A1).

### Awareness of government-issued guidelines on antibiotics use

When asked about awareness of government-issued guidelines on antibiotic use in animals at either national, regional, state or local levels, 217/384 (56.5%) respondents said ‘No’ to having encountered any guidelines whilst 107/384 (27.9%) said ‘Yes’ as they had encountered such guidelines at one of the aforementioned levels. A further 60/384 (15.6%) responded that they ‘don’t know’ if they had encountered such documents or not (Appendix 1 – Table 7-A1). The government-issued guidelines seen by the 107 respondents were in the form of leaflets (49/107 [45.8%]), brochures (40/107 [37.4%]), reports (59/107 [55.1%]) and radio announcements (12/107 [11.2%]). Other reported sources including animal disease act, Internet, drug formula information, seminars, VCN drug formulary, and public health forums accounted for another 10/107 (9.3%) of respondents (Appendix 1 – Table 7-A1). Of the 107 (107/384 [27.9%]) respondents who had knowledge of government-issued guidelines on antibiotic use, 28/107 (26.2%) had never used these guidelines to inform their decision-making on antibiotic use whilst another 74/107 (69.2%) used the issued guidelines. A further 5/107 (4.7%) were unsure (Appendix 1 – Table 7-A1).

Lastly, amongst the 384 survey respondents who recommended the use of antibiotics, 311/384 (81%) of these believed government-issued legislation or regulation on antibiotic use in animals can influence the use of antibiotics by veterinarians, para-veterinarians and other animal health professionals. Fifty-six (56/384 [14.6%]) believed guidelines and legislation would have no effect on habits of animal health practitioners, whilst a further 17/384 (4.4%) were unsure (Appendix 1 – Table 7-A1).

## Discussion

This study provides some insights into the antibiotic use practices of veterinarians and para-veterinarians in Nigeria. Most of the respondents were young veterinarians who worked in private practice and had less than 10 years practice experience. These survey demographics may be attributed to how the survey questionnaire was distributed, predominantly via social media groups (such as WhatsApp groups) of veterinarians across Nigeria. Younger people are more likely to be aware of information technology and more commonly use social media platforms (Vaportzis, Giatsi Clausen & Gow 2017). There has also been an increase in the number of graduating veterinarians lately because of an increasing number of established veterinary faculties within the last 15 years including at the University of Jos (Plateau State), the University of Abuja (Federal Capital Territory), the University of Ilorin (Kwara State), and Michael Okpara University of Agriculture (Umudike, Abia State). Because young people made up the bulk of the animal health practitioners who participated in this survey, responses could therefore have been inherently biased towards highlighting the antibiotic use practices of younger veterinarians and para-veterinarians.

Most of the respondents recommended the use of antibiotics in animal patients, principally targeting bacterial pathogens. However, a high proportion of respondents also recommended the use of antibiotics to manage non-bacterial pathogens, including viruses, protozoans and even fungi. By using antibiotics indiscriminately for the management of non-bacterial pathogens such as viruses and fungi, additional bacteria are exposed to selection pressure, destroying commensal but antibiotic-sensitive microflora, allowing resistant strains to proliferate and leading to increased risk of AMR (Ventola 2015). Some antibiotics are indicated in the effective management of protozoan parasites (Sainz et al. 2015); although aside these few exceptions, antibiotics should be exclusively used for bacterial disease treatment. The reported incorrect use of antibiotics by veterinary professionals suggests a lack of knowledge on appropriate antibiotic use practices. In this study, it is also possible that the prescription of antibiotics for non-bacterial pathogens may have been driven by non-clinical issues because they too are important considerations for veterinarians. The non-clinical factors reported to influence the decision to prescribe antibiotics include a farmer preference or request to have antibiotics administered (Gibbons et al. 2013; McDougall, Compton & Botha 2017), fear of being blamed if it is later proved that antibiotics were required (Gibbons et al. 2013) and withholding period (McDougall et al. 2017). In Nigeria, there are no guidelines restricting access to veterinary drugs. Farmers, veterinarians and para-veterinarians have access to all veterinary antibiotics from veterinary practices, agro-shops and even open drug markets. As such it is difficult to know who prescribed what, when and the amounts. A dearth of veterinary professionals and dysfunctional veterinary systems mean that non-veterinary professionals, although not licensed to, often engage in disease diagnosis and treatment, thus increasing the chance for misdiagnosis and antibiotic misuse. Whilst in most developed countries, only registered veterinary practitioners can administer certain class of antibiotics, government officials do not usually administer antibiotics and para-professionals are also not allowed to prescribe. The situation in Nigeria underlines the importance of adopting and enforcing regulation aimed at reducing inappropriate antibiotics use such as classifying antibiotics as prescription only drugs, establishing strict guidelines for appropriate antibiotic use and limiting access to professionals only.

In this survey a low frequency of antimicrobial susceptibility testing (AST) use was reported with over 70% of respondents either very sparingly conducting AST prior to antibiotic treatment or not at all. Findings in this study contrast with those reported elsewhere. A study carried out in Washington State, United States of America, reported that majority of respondent veterinarians (166/203, 82%) were engaged in small animal or exotic animal practice, and 76% ordered AST regularly in their practice and 24% did not order AST at all (Fowler et al. 2016). In another study carried out across Europe, 44.3% of veterinarians carried out AST when cases were unresponsive or complicated, 24.3% regularly used AST testing and 9.8% of veterinarians never used AST to inform antibiotic prescription (De Briyne et al. 2013). And in a study conducted in New Zealand, culture and AST was not widely used (McDougall et al. 2017). In the three studies mentioned, the type of veterinary practice for respondents who did not use AST was not specifically reported, to allow for an assessment of the potential link between low AST use and the type of veterinary practice. It is important to note that AST is not always required in practice as clinicians frequently make diagnosis and treatment decisions based on empirical knowledge; empirical antibiotic treatment can be started whilst awaiting AST results and such treatment can be changed once AST results become available if appropriate. The use of AST where applicable can help eliminate uncertainty and provide guidance on the selection of an effective antibiotic as the first-line treatment and can subsequently contribute to reduced antibiotic consumption overall. However, there is a potential downside to the use of culture and AST, which is the inevitable delay between sampling and obtaining a result, which may take at least 24 h – 48 h with resultant delaying of onset of treatment that may affect the outcome. Other key constraints to AST use have been reported in Nigeria and elsewhere, and these include limited availability and access to laboratories with capacity to conduct culture and AST and affordability of the associated costs (Adekanye et al. 2020; Fowler et al. 2016). Addressing the barriers to AST use may help increase AST use and promote appropriate antibiotic use.

Findings of this study revealed that a large proportion of the respondents used laboratories only sparingly or not at all. The question on the use of laboratories was added to this study because in most sub-Saharan Africa countries, AST is primarily performed in laboratory settings and as such, its conduct is intrinsically linked to the availability and access to laboratory facilities. The low use of laboratories for diagnosis in this study may be linked to a shortage of laboratory diagnostic facilities. The unavailability of veterinary laboratory services and the owner’s inability to pay have been reported as barriers to conducting AST in Nigeria (Adekanye et al. 2020), and the shortage of laboratory diagnostic facilities has also been reported elsewhere in sub-Saharan Africa (Nakayima et al. 2016). The existing animal health laboratories and a host of emerging laboratories are mostly restricted to the 11 veterinary teaching hospitals in Nigeria and the National reference veterinary laboratory in Vom, Plateau State. In addition to the institutional laboratories mentioned here, several other private laboratories have recently been established, either owned by companies or private veterinary clinics. For example, Animal Care, a leading veterinary animal health company in Nigeria, has a network of six laboratories located across the country. Despite this array of existing laboratories and a host of emerging laboratories, the geographically expansive nature of Nigeria suggests that existing laboratories may be insufficient to cater for the diagnostic needs of veterinarians. Even where laboratories are available, logistics challenges such as access and transportation, as well as lengthy laboratory turnaround time, and the absence of a quality assurance system to regulate veterinary laboratories further limits patronage of existing laboratories. Creating a good laboratory network with capability to perform procedures such as AST would assist veterinary and para-veterinary practitioners in making informed choices on antibiotic use and support clinical and diagnostic decisions. Through enhanced collaborations and communication between animal health professionals and laboratory personnel, animal health practitioners can also keep abreast with disease epidemiology and trends.

The survey findings indicated that chickens were the most frequently cited species cared for by respondents and the species for which antibiotics were mostly used. More than 50% of respondents also reported using antibiotics in ruminants and dogs compared with cats, turkeys or aquaculture. Identifying species and indications for which most antibiotics are used may be beneficial in highlighting potential areas to be focussed on to improve appropriate antibiotics use and reduce the risk for AMR emergence.

Oxytetracycline and tylosin were reported as the most frequently recommended antibiotics to treat animals by the respondents in the present study. This is consistent with the studies conducted in Nigeria and elsewhere. Alhaji and Isola (2018) reported oxytetracycline, tylosin and penicillin as the most widely used antibiotic in north central Nigeria, and another study in Europe reported tetracycline and penicillin as the most frequently prescribed classes of antibiotics (De Briyne et al. 2014). The high use of oxytetracycline may contribute to the frequently documented resistance levels of tetracyclines in animals (Oloso et al. 2018). Similarly, tetracycline residues have been identified as the most common antibiotic residues in locally produced poultry and cattle, in contrast with the low levels found in imported poultry products (Oloso et al. 2018). Incidence patterns of AMR in human enteric *E. coli* showed a high level of tetracycline resistance in Nigeria, suggesting a basis for further investigation into the relationship between oxytetracycline use in animals and emerging tetracycline resistance in humans (NCDC 2019). Enrofloxacin was amongst the most frequently reported prescribed antibiotics in this study; over a third (38.5%) of the respondents reported prescribing enrofloxacin in animals. Fluoroquinolones are categorised as critically important antimicrobials (CIAs) for human health (Erwin et al. 2020; WHO 2019), and there is concern that the use of enrofloxacin in animals could lead to sharing of genes encoding resistance to fluoroquinolones in people. Veterinarians can play a role in protecting CIAs (Wood 2018) such as enrofloxacin by using these as ‘last resort’ antimicrobials. In so doing, veterinarians would contribute to tackling the problem of AMR in both animals and humans.

Disease treatment was reported as the main reason for recommending the use of antibiotics to treat animals. This finding is expected and may be attributed to the survey respondents being veterinarians and para-veterinarians with academic and practical knowledge on appropriate use of veterinary pharmaceuticals gained from veterinary training and education. A considerable proportion of respondents (40%) reported recommending the use of antibiotics for disease prevention or prophylaxis, but details on the type of prophylaxis were not collected. Further investigation on the type of prophylaxis is necessary to determine if the use of antibiotics was appropriate and to inform the creation of relevant guidelines. For example, if the use of antibiotics for disease prevention or prophylaxis was inappropriate, local government regulation could be enforced to encourage appropriate antibiotic use. The presence of regulatory controls would align with other countries, where the use of antibiotics for disease prevention, and as growth promoters, is restricted and controlled (Kirchhelle 2018; More 2020; Tang et al. 2017). This is seen in countries such as the Netherlands (Mevius & Heederik 2014; Speksnijder et al. 2014) and the United Kingdom (NOAH 2020).

Veterinary clinics, agro-shops and open markets are the traditional sources of licensed veterinary drugs in Nigeria. Other potential sources, for example online pharmacies and drug compounding pharmacies, although used in human medical practice on a limited scale, remain uncommon in veterinary practice. Although most animal health practitioners reported obtaining antibiotics from veterinary clinics, some reported purchasing antibiotics from open markets. Sourcing of antibiotics from open markets is of concern as these sources provide greater unregulated access to all classes of antibiotics by non-professionals compared with veterinary clinics, agro-stores and distributors. For example, veterinary drug distributors found in open markets are less likely to offer buyers any technical advice on proper antibiotic use. Products from open markets are mostly unregulated, less likely to be registered and more likely to have expiry dates altered. Such products are also less likely to contain product leaflets to guide proper dosing and adherence to withdrawal periods. This is in contrast to getting antibiotics from veterinarians and agro-stores, who are more likely to adhere to regulations, ask questions and query the intended use and possibly make recommendations. It is additionally important to note that whilst the open market may not enjoy significant patronage from professionals, there is considerable drug movement between veterinary clinics, agro-shops and distributors. For instance, veterinary clinics may readily source drugs from agro-shops and distributors whilst agro-shops in turn may access from both the clinics and distributors. Despite being regarded as common occurrence, managing of drugs by non-professionals brings a new dimension to AMR. In an open market environment, drug storage can play a huge role in drug potency with hot and humid conditions degrading medications, reducing drug effectiveness and potency (Ocan et al. 2014). The sub-therapeutic doses resulting from drug degradation may lead to repeated antibiotic treatment and use and to increased selection pressure and survival of the most resistant organisms. Beside poor storage, drug adulteration and counterfeit drugs (Akiny 2013) also appear to be risks in open unregulated markets. This highlights the need for government regulation, enforcement and penalties aimed at promoting AMS.

Most survey respondents consulted manufacturer labels on drug bottles, sachets or leaflets for guidance on antibiotic choice and use. Personal experience also played a substantial role in antibiotic choice and use, in addition to consulting colleagues or following guidelines developed by individual practices. Two studies carried out in Europe also reported personal experience as one of the top factors influencing antibiotic choice (Coyne et al. 2018; De Briyne et al. 2013). Although often useful, personal experience may sometimes provide subjective views on antibiotic prescription habits; potentially based upon outdated knowledge and practices. Similarly, colleague guidance cannot always be guaranteed as a reliable and current source of information. Whilst personal experience and manufacturer guides are vital, they should be supported by laboratory results (Watts, Sweeney & Lubbers 2018). Previously potent antibiotics can now be ineffective against once treatable bacteria, with such cases only verifiable by using diagnostic support. Reliance on prior experience and product labels alone may lead to unsuccessful treatment decisions, contributing to inappropriate and repeated antibiotic use, thus increasing the risk for AMR development.

Many of the respondents were unaware of the existence of any formally issued regulatory guidelines on antibiotic use in Nigeria. Collectively, almost all participants agreed that government-issued legislation or guidelines, if properly publicised, would influence the ways in which antibiotics are used by animal health workers. The lack of knowledge of formally issued regulatory guidelines on antibiotic use in this study is not surprising as there are no documented systematic trainings or defined methods for dissemination of this information to practising and newly qualified animal health professionals by regulatory authorities. However, there is a Nigerian government action plan to tackle the threat of AMR (FMARD 2017). A quarter of the respondents who reported in this study were aware of government-issued guidelines on antibiotic use in animals; it is possible these respondents might have encountered guidelines that originated from sources other than the government. Alternatively, those respondents might have provided incorrect responses. The introduction and use of guidelines were reported to lead to appropriate use of antimicrobials by small animal clinicians in the United Kingdom (Hughes et al. 2012). It is therefore possible that the provision of such documentation by bodies including the National Agency for Food and Drug Administration and Control (NAFDAC; responsible for drug regulation in Nigeria), government ministries (such as the Federal and State Ministries of Agriculture and Rural Development), private practices and veterinary institutions may promote the use of reliable sources of information on appropriate antibiotic use by animal health professionals in Nigeria.

To be effective, guidelines should be supported by relevant scientific literature and be periodically revised with updated information. In addition, however, a balanced pragmatic approach to antibiotic regulation should also consider the needs of farmers and how they may be impacted by establishing certain antibiotic restrictions. This will ensure that farmers and animal health workers alike have the knowledge and treatments necessary to deal with prevalent diseases whilst simultaneously promoting prudent antibiotic use.

In general, the study’s findings suggest that a number of additional strategies can be explored in an effort to improve AMS. Training and education of farmers, veterinarians and para-veterinary professionals on adherence to drug withdrawal periods and adoption of vaccination can play an important role in reducing the risk of AMR. The education of both veterinary professionals and farmers on drug withdrawal periods may help reduce the risk of consumption of products (such as eggs, milk and meat) by the consumers that include antimicrobial residues. Improved management practices including the adoption of vaccination can prevent and help reduce disease burden and subsequently the amounts of antibiotics used for treatments. Having a closer working relation between veterinary professionals and animal owners can also contribute positively towards disease prevention. And finally, establishing effective laboratory quality control systems combined with effective support of laboratory service delivery from key players such as the government and industry may also contribute towards positive antibiotic stewardship.

A few limitations were observed in this study. Firstly, the survey had no means of definitively validating the identities of the respondents, and it was difficult to conduct an analysis to determine if differences existed between responders and non-responders because no information was obtained on non-responders. To ensure the correct target audience was reached, the survey link was shared exclusively with veterinarians and para-veterinarians and only professional chat groups were targeted.

Secondly, participation may have been limited by a lack of access to the Internet and ownership of an electronic device. The survey required both access to the Internet and ownership of an electronic device (android or iOS phone, tablet, computer, etc.) on which to complete the survey. An unknown proportion of veterinarians and para-veterinarians would not meet these criteria and consequently were unable to participate. Participation may also have been limited by how the veterinarians and para-veterinarians who were active on social groups were identified. Identification of the social groups to which a survey link was sent depended in part on the number of contacts made by authors with different key association officials and KOLs as well as on authors’ personal knowledge. It is possible that this approach may not have been broad enough to capture all veterinarians who are active on social media nationally.

Thirdly, the investigators could not determine how many respondents completed the survey via phones versus tablets versus laptops and could not determine the response rate per chat group or channel used (i.e. how many respondents were got from each of these individual channels) to distribute the survey link.

Fourthly, the study may have also been subject to participant selection bias as survey distribution was dependent on social circles and networks. This may have resulted in distribution being biased or restricted to the groups and people the study team was aware of (e.g. inclusion of more Nigeria Veterinary Medical Association groups compared with Paravet association groups), unconsciously omitting other unknown social media groups. Furthermore, some veterinarians may be active on the Internet but are either not currently practising or are working in alternative sectors with little or no relationship with those in the veterinary industry. Nonetheless, these individuals could still use antibiotics internally on private farms or even on their pets. Consequently, certain categories of potential respondents may have been inadvertently excluded from this study, impacting on the inclination of results generated.

In addition, some of the responses may have been impacted by a recall bias. Veterinarians and para-veterinarians were asked to respond to survey questions by recalling information from practice experience and general knowledge. Respondents may be impacted by memory losses or an inability to remember accurately.

Also, some of the antibiotics in the survey questionnaire were listed as trade names. Although this was done to accommodate common antibiotic combinations present in the market and in use, it is possible that not every respondent would be familiar with specific trade names, and this might have impacted negatively on the objectivity of responses.

Another related limitation is that data on antibiotic type and use were not collected on a species basis. Understanding antibiotic choice at species level and the related diagnostic support sought may provide useful insights to help understand areas for improvement that should be targeted.

Education on antimicrobial use in veterinary school was not added as an option when assessing sources of information used to guide decisions on choice of antibiotics. This is an important source of information especially for new graduating veterinarians entering clinical practice.

Finally, total participant demographics show that younger veterinarians and para-veterinarians made up the bulk of the respondents. This may be characteristic of how younger people tend to be more active than older people on social media platforms and be more competent in the requisite skills to use android, iOS or laptop devices. Although assumed to be less influential in electronic survey completion compared with face-to-face survey conduct, the social desirability bias may have also affected survey responses. The social desirability bias pertains to the behaviour of research participants to select responses based on perceived socially desirable answers, rather than providing the most truthful response (Grimm, 2010). In the context of this study, some survey respondents may have declined to share antibiotic-prescribing habits they considered inappropriate or erroneous, resulting in an under-reporting of certain antibiotic usage behaviours.

## Conclusion

The survey’s findings suggest promoting AST use and addressing the potential constraints to AST use such as limited availability and access to laboratories with capability to conduct culture and AST, and affordability of the associated costs may help improve adoption and use of AST.

Findings also suggest that there is an inappropriate use of antibiotics for the management of non-bacterial pathogens such as fungi and viruses. Establishing strict guidelines for appropriate antibiotic use and limiting access to antibiotics to professionals only can be considered in regulation aimed at reducing inappropriate antibiotic use.

The inadequacies in the availability of and adherence to publicised regulatory guidelines on antibiotic use indicate a need for regulatory authorities to urgently address the creation and wide circulation of legislation and guidelines for use by animal health practitioners. The effectiveness of formally issued guidelines will be highly dependent on the breadth of their distribution across the country and their continued promotion by authoritative bodies.

## Appendix 1

For some questions, survey respondents were invited to select all applicable responses and as such the respondent’s total may not add up to 100% for these variables. For clarity, these questions are labelled as ‘All responses selected’.

**TABLE 1-A1:** Animal species most commonly seen in practice (*n* = 384).

Animal species	Respondents†	
	*n*	%
Chickens	260	67.7
Dogs	251	65.4
Goats	197	51.3
Sheep	178	46.4
Cattle	176	45.8
Cats	94	24.5
Turkeys	82	21.4
Fish/Aquaculture	61	15.9
Rabbits	52	13.5
Exotics	29	7.6
Others	11	2.9

†, All applicable responses selected.

**TABLE 2-A1:** Most commonly prescribed antibiotics in practice (*n* = 384).

Antibiotics	Respondents†	
	*n*	%
Oxytetracycline	317	82.6
Tylosin	171	44.5
Gentamycin	168	43.8
Penicillin	151	39.3
Enrofloxacin	148	38.5
Neoceryl/Keproceryl® (Neomycin, Erythromycin, Oxytetracycline, Streptomycin and Colistin)	141	36.7
Metronidazole	111	28.9
Amoxicillin	93	24.2
Doxycycline	77	20.1
Streptomycin	74	19.3
Ciprofloxacin	73	19.0
Tetracycline	65	16.9
Doxy-get (Doxycycline, Gentamycin)	60	15.6
Neomycin	34	8.9
Neomycin, Chloramphenicol and Oxytetracycline (NCO)	26	6.8
Ampicillin	13	3.4
Furazolidone	7	1.8
Flumequine	7	1.8
Others	4	1.0
Norfloxacin	1	0.3

† , Respondents select top five.

**TABLE 3-A1:** Type of pathogen targeted when prescribing antibiotics to animals (*n* = 384).

Pathogen	Respondents†	
	*n*	%
Bacteria	379	98.7
Viral	316	82.3
Protozoal	312	81.3
Helminth (worms)	273	71
Fungal	265	69
Others	5	59.3

† , All applicable responses selected.

**TABLE 4-A1:** Most common sources of antibiotics used in practice (*n* = 384).

Source	Respondents†	
	*n*	%
Veterinary clinic (veterinarian)	259	67.4
Agro-store (non-veterinarian)	177	46.1
Distributor	154	40.1
Open markets	60	15.6
Others	7	1.8

† , Two most applicable responses selected.

**TABLE 5-A1:** Sources of information most commonly used to guide decisions on antibiotic use (*n* = 384).

Source	Respondents†	
	*n*	%
Manufacturer labels found on bottle, sachet or leaflet	289	75.3
Personal experience	278	72.4
Antibiotics guidelines developed and kept by my practice	195	50.8
Consulting colleagues	193	50.3
Others	13	3.4

† , Three most applicable responses selected.

**TABLE 6-A1:** Common types of tests requested when submitting samples for laboratory testing (*n* = 384).

Test	Respondents†	
	*n*	%
Microbiological culture and sensitivity testing	236	61.5
Pathology/post-mortem examination	168	43.8
Microbiological culture, excluding sensitivity testing	102	26.6
Do not submit samples	79	20.6
Serology	73	19.0
Others	7	1.8

† , All applicable responses selected.

**TABLE 7-A1:** Awareness, formats encountered, use and opinions on Nigeria government-issued guidelines on antibiotic use in animals.

Variable	Response	*n*	%
Awareness of government-issued guidelines on antibiotic use in animals (*n* = 384)	No	217	56.5
	Yes	107	27.9
	Don’t know	60	15.6
Format of government-issued guidelines on antibiotic use in animals encountered† (By respondents aware of guidelines, *n* = 107)	Reports	59	55.1
	Leaflets	49	45.8
	Brochures	40	37.4
	Radio announcements	12	11.2
	Other	10	9.3
Use of government-issued guidelines to support decision-making on antibiotic use in practice (By respondents aware of guidelines, *n* = 107)	Yes	74	69.2
	No	28	26.2
	Don’t know	5	4.7
Opinion on statement ‘Government regulation or legislation on antibiotic use in animals can influence the use of antibiotics by animal health professionals’ (*n* = 384)	Agree	311	81.0
	Disagree	56	14.6
	Don’t know	17	4.4

† , All applicable responses selected.
